# Diabetic retinopathy: research to clinical practice

**DOI:** 10.1186/s40842-017-0047-y

**Published:** 2017-10-19

**Authors:** Anjali R. Shah, Thomas W. Gardner

**Affiliations:** 1 Departments of Ophthalmology and Visual Sciences, University of Michigan Medical Schoo, W.K. Kellogg Eye Center, 1000 Wall St, Ann Arbor, MI 48105 USA; 2 Molecular and Integrative Physiology, University of Michigan Medical School, W.K. Kellogg Eye Center, 1000 Wall St, Ann Arbor, MI 48105 USA

**Keywords:** Diabetic retinopathy

## Abstract

**Background:**

Diabetic Retinopathy (DR) is a leading cause of visual impairment in the United States. The CDC estimates that the prevalence of DR will triple from 2005 to 2050.

**Main body:**

The report summarizes major past advances in diabetes research and their impact on clinical practice. Current paradigms and future directions are also discussed.

**Conclusions:**

DR is a leading cause of visual impairment in the US. Significant progress has been made in the understanding and treatment of DR, but rising prevalence demands innovative approaches to management in the future.

## Background

Diabetic retinopathy (DR) is the ocular manifestation of end-organ damage in diabetes mellitus. Eduard Jaeger first described the visible retinal changes of DR in 1856, but the causal relationship between retinal exam findings and diabetes mellitus was controversial until 1875 when Leber confirmed the findings [1]. Today, DR is a leading cause of visual impairment in the United States. In 2005, 5.5 million people had diabetic retinopathy, and 1.2 million people had vision-threatening DR. Due, in large part, to the projected increase in prevalence of diabetes mellitus, the CDC projects that by 2050 those numbers will triple, to 16.0 million and 3.4 million, respectively (https://www.cdc.gov/visionhealth/publications/diabetic_retinopathy.htm). Fortunately, a better understanding of the risk factors contributing to the development of DR, the pathology of the disease, and its functional manifestations have allowed for significant advances in the prevention and treatment of diabetic retinopathy. This review presents the contributions of research to the clinical management of the disease in the past, discuss current paradigms on DR treatment and prevention, and demonstrate how today’s research will contribute to improved outcomes in the future.

## Diabetic retinopathy: Research to clinical practice—Past

Since the earliest description of DR, the vascular features of the disease have been predominant. Early drawings show intraretinal hemorrhages, vascular sheathing and lipid exudates throughout the retina. These findings were confirmed with histopathological specimens, such as the work of Arthur Ballantyne, who, in 1945, showed that capillary wall changes contributed to the development of DR [1]. Laboratory research on endothelial cell dysfunction and clinical observations using fluorescein angiography solidified the paradigm of DR as a vascular disease, and led to early suggestions of using light photocoagulation, or laser therapy, to treat retinopathy in the 1960s [2]. In 1968, the Airlie House Symposium brought prominent ophthalmologists and researchers in diabetic retinopathy together. It was during this meeting that a standard classification system for diabetic retinopathy was created, and the foundation was set for future large clinical trials.

The Diabetic Retinopathy Study (DRS) and Early Treatment of Diabetic Retinopathy Study (ETDRS), conducted in the 1970s and 1980s, respectively, demonstrated the considerable effects of laser treatment in eyes with proliferative retinopathy and macular edema. The DRS found that pan-retinal photocoagulation (PRP) inhibited the progression of retinopathy in patients with proliferative (neovascular) changes [3]. ETDRS defined “clinically significant macular edema” and demonstrated that focal photocoagulation significantly reduces the risk of vision loss from diabetic macular edema [4]. As a result of these landmark trials, PRP and focal laser became standard care for patients with advanced diabetic retinal disease in the 1980’s, and, despite the success of intravitreal injection therapy, continue to be frequently performed procedures today. These studies also led to the development of guidelines and screening programs to allow timely detection and treatment of DR.

In addition to making strides in the treatment of diabetic retinopathy, the latter half of the twentieth century saw major advances in understanding the risk factors leading to development and progression of disease. The importance of tight metabolic control wasn’t unequivocally demonstrated until 1993 when the Diabetes Control and Complications Trial (DCCT) followed type 1 diabetic patients with mild or no retinopathy for a mean of 6.5 years, and found that intensive insulin therapy reduced the adjusted mean risk for development of retinopathy by as much as 76% [5]. Similar results were noted in persons with type 2 diabetes by the United Kingdom Prospective Diabetes Study (UKPDS) Group; intensive control of blood sugar led to a significant 25% reduction in the risk of any microvascular complications, including retinopathy, nephropathy and neuropathy. Most of this decrease in risk, however, was attributed to decreased need for laser in proliferative retinopathy. Overall, there was a 21% reduction in the risk of progression of DR [6].

The role of hypertension in the development and progression of diabetic complications was also demonstrated initially in 1998 by UKPDS [7]. Tight blood pressure control (defined as <150/85 mmHg) achieved a 34% reduction in the rate of progression of DR, independent of glycemic control after 7.5 years. These findings were subsequently supported by several studies showing that blood pressure management significantly reduces the risk of progression of DR [8, 9]. As a result of better systemic management of diabetes mellitus and hypertension, as well as the development of screening programs and improved, more timely treatment, the incidence of proliferative DR and/or diabetic macular has decreased significantly, especially in type 1 diabetics. From 1980 to 2007, the Wisconsin Epidemiologic Study of Diabetic Retinopathy (WESDR) showed a 77% decrease in the estimated annual incidence of proliferative DR among persons with type 1 diabetes, and the incidence of visual impairment due to diabetic retinopathy decreased by 57% in that same time period [10, 11]. The prevalence of DR in persons with type 2 diabetes aged 40 or older in WESDR (1980–1982) was 50%, and 35% in the Beaver Dam Eye Study (1988–1990), suggesting a substantial decrease in prevalence of DR during the interval 8 year period. This reduction may be attributed to better overall medical care of patients with diabetes [12].

The translation of research to clinical practice in the twentieth century is a story of great achievement—both for individuals suffering from diabetes and its complications, and on the public health front. In 1992, it was estimated that the Diabetic Retinopathy Study alone, which cost $10.5 million to conduct, generated a net savings of $2.89 billion to society in the first decade after the trial was completed [13].

## Diabetic retinopathy: Research to clinical practice—Present

The success of clinical and epidemiological research of the 1970s, 80s and 90s encouraged further research into risk factors for development and progression of disease. In addition to hyperglycemia and hypertension, large randomized clinical trials showed the efficacy of lowering serum lipid levels. Notably, fenofibrate was shown to reduce the need for laser photocoagulation [9, 14]. In conjunction with simvastatin, fenofibrate reduced the risk of progression of non-proliferative retinopathy by one-third [9], though fenofibrate did not reduce cardiovascular risk [15]. Despite this sizable effect, fenofibrate therapy for DR has not become standard practice. Reasons for deferred incorporation of fenofibrate into routine clinical practice are unclear and likely multifactorial. In addition to a lack of clarity on when and how to use the medication, the lack of a commercial sponsor may also play a role. Additional risk factors for progression of DR discovered were the presence of nephropathy [16] and pregnancy [17]. Thus, comprehensive systemic care of persons with diabetes is required to achieve optimal vision.

Though focal laser and PRP reduce the risk of vision loss and DR progression, there are several limitations to photocoagulation. Laser is primarily a destructive procedure, such that PRP may impair peripheral vision, and decrease night vision [18]. In addition, focal laser seldom actually improves visual acuity [19]. Thus, more effective treatments for the early and late stages of DR are needed.

The paradigm of DR as a primarily vascular disease was pursued further. Several studies confirmed that neuro-inflammation plays a prominent role in the pathogenesis of DR [20–22]. Steroids, delivered intravitreally, are effective in improving vision in patients with diabetic macular edema, though they cause cataract development and increased intraocular pressure leading to glaucoma [23, 24]. Intravitreal steroids have also been suggested to reduce the rate of progression of DR to proliferative disease as well [25, 26].

Increased levels of inflammatory mediators ultimately lead to breakdown of the blood-retinal-barrier, increased vascular permeability, and angiogenesis via the release of cytokines and growth factors, including vascular endothelial growth factor (VEGF) [27–29]. The resulting new pharmacotherapeutic targets led to significant changes in the management of patients with DR, and the development of a new standard of care.

Several clinical trials showed the efficacy of intravitreal anti-VEGF medication in the treatment of diabetic macular edema [30–32]. These studies confirmed that administration of repeated monthly intravitreal ranibizumab injections (an anti-VEGF medication) plus prompt or deferred laser yielded a modestly greater improvement in visual acuity from baseline than laser alone with minimal side effects, leading to the adoption of a new standard of care in the treatment of diabetic macular edema. In addition, progression of DR can also be slowed in 25–30% of eyes treated for 2 years with the use of anti-VEGF medications [33]. It is interesting to note that despite the decrease in progression noted in 25–30% of treated eyes, the majority of patients did not demonstrate such positive effects. These results suggest that there are factors in addition to VEGF that likely mediate DR progression. A large randomized clinical trial showed that an average of nine intravitreal injections of ranibizumab was non-inferior to PRP at 2 years, with fewer patients who received ranibizumab requiring vitrectomy surgery over the study period [34].

**Fig. 1:**
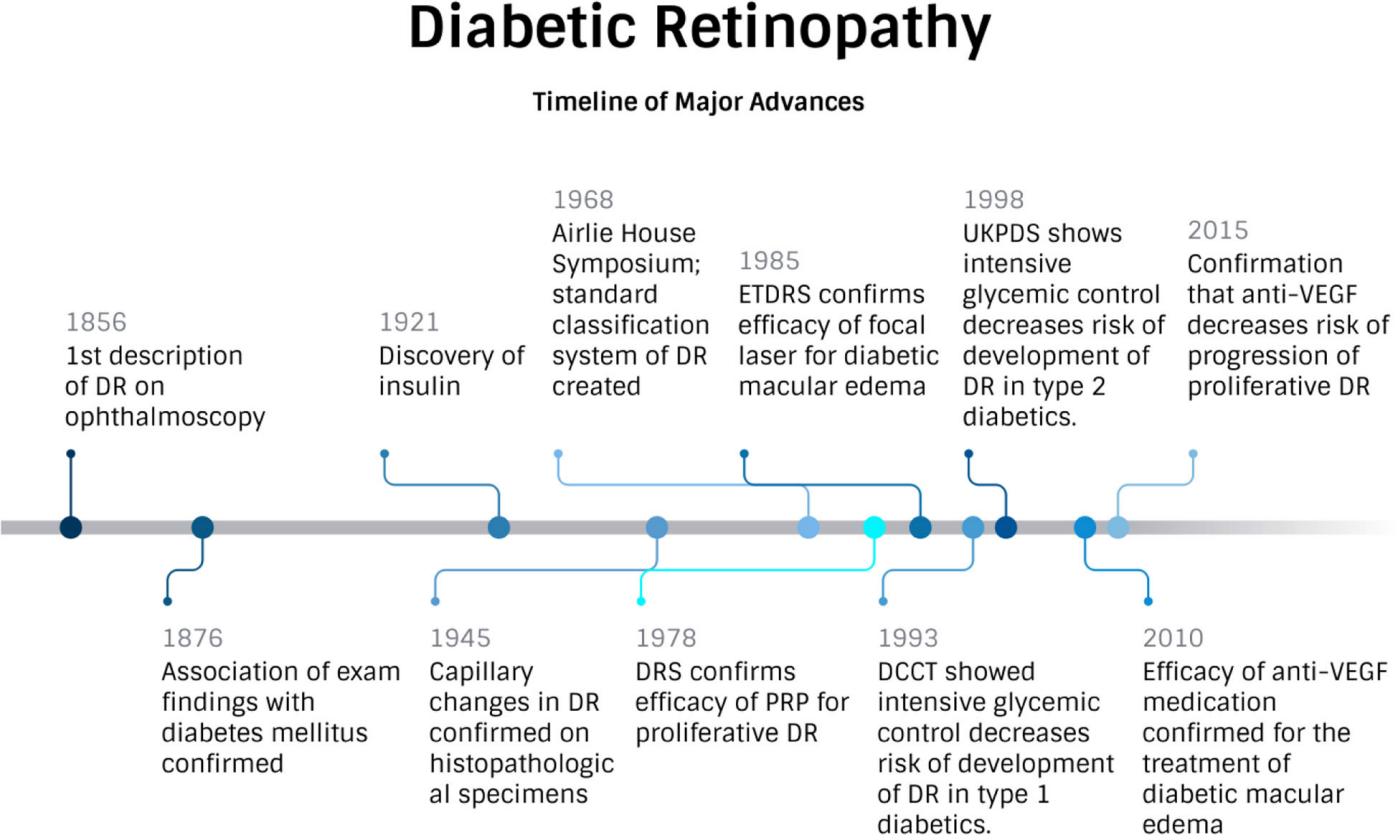
Diabetic Retinopathy: Timeline of Major Advances

Research on the role of inflammation in DR, and the subsequent increase in vascular permeability led to significant changes in the way patients have been managed in the past decade. Anti-VEGF therapy for diabetic macular edema has been shown in multiple randomized clinical trials to be more effective at improving vision than laser, and several cost-effectiveness analyses have confirmed the value of these treatments to patients and society [35, 36]. The efficacy of anti-VEGF treatment has also been proven in the treatment of proliferative DR and use of these medications in the management of this condition has increased, often as a first line treatment for complications of proliferative DR, such as vitreous hemorrhage. Other factors, however, have prevented wide-spread adoption of the practice as standard of care. For optimum results, anti-VEGF medications demand frequent administration—potentially indefinitely—resulting in concerns about the overall cost of treatment and the increased burden placed on physicians and patients by the need for frequent, consistent follow up to maintain treatment gains. Intravitreal anti-VEGF therapy does offer, though, a viable adjuvant or alternative treatment in many cases of proliferative disease [37]. Thus, we now have surgical (laser) and pharmacologic (anti-VEGF) options to treat DR and patients and many physicians tailor these approaches, using them separately or together, to optimize benefits and convenience. Figure 1 depicts the timeline of major advances in diabetic retinopathy research to date.

This significant progress in management of diabetic retinopathy was coupled with, and made possible by, important developments in ocular imaging [38]. Optical coherence tomography (OCT) is a non-invasive diagnostic test that is performed in the office, providing detailed cross-sectional anatomic images of the retina. In its widest application, OCT allows for early detection of anatomical changes in the macula, such as the development of thickening and cystic spaces noted in diabetic macular edema. OCT testing is routinely used to clinically diagnose and manage patients with diabetic macular edema, and data on central retinal thickness from the OCT is used as an end-point in large clinical trials [39]. Additional imaging techniques such as ultra-wide-field fundus photography and angiography allow better visualization of the peripheral retina than conventional cameras, and better identification of areas of poor vascular perfusion [40]. These imaging modalities help with clinical management of patients, and provide further insight into structural changes in every stage of DR.

## Diabetic retinopathy: Research to clinical practice-future

The progress of the late 20^th^ and early 21^st^ centuries has been significant, allowing improved ability to delay development of DR, and to administer treatment that significantly reduces the risk of vision loss. However, the large projected increase in prevalence of DR, coupled with the need for frequent administration of intravitreal injections clearly indicates a need for alternative options in the future. In addition, current management strategies are either preventative (intensive glycemic and blood pressure control), or targeted towards advanced disease (diabetic macular edema, or proliferative DR). Yet, a growing body of literature suggests functional decline and associated health-related quality of life reductions in earlier stages of DR [41]. Visual dysfunction in the form of decreased sensitivity on visual field testing and diminished photoreceptor function as measured by electroretinogram have been reported prior to the development of vascular lesions [42, 43].

Thus, the paradigm on DR has changed. Alterations in the neurosensory retina, undetectable by ophthalmoscopy, are recognized as important early contributors to visual decline, and it is now established that neurosensory degeneration may precede visible vascular changes, or occur alongside them [44]. That is, the entire neurovascular unit, comprised of vascular, glial, microglial and neuronal cells, is compromised by diabetes [45]. When the neurovascular unit is no longer intact and adaptive processes fail after years of uncontrolled diabetes, the states of diabetic macular edema and proliferative retinopathy represent “retinal failure,” equivalent to renal failure [46]. Current treatments of anti-VEGF and lasers address these late stages of disease, but only fenofibrate, blood pressure and metabolic control have shown demonstrable effects in the pre-failure stages.

Laboratory research confirms that metabolic pathways triggered by hyperglycemia, insulin deficiency [47] and dyslipidemia [48] lead to abnormalities in both the neural retina as well as the retinal capillary bed. A better understanding of all the molecular players in these pathways has produced several potential pharmacotherapeutic targets for DR, which includes both the inhibition of mediators of neural damage, and enhancement of agents that may be neuroprotective. Hernández et al. [49] recently provided a thorough review of potential new therapeutics based on pathogenic mechanisms of DR, much of which is summarized in Table 1.

**Table 1:** Potential Therapeutics for Diabetic Retinopathy

Target	Role	Current Status	Concerns
Somatostatin	Neuroprotective, antiangiogenic. Downregulated in retinas of diabetics, associated with retinal neurodegeneration [54].	Recently completed multi-center phase II-III trial (EUROCONDOR) to assess the safety of topically administered somatostatin. (EudraCT Number: 2012–001200-38) Results have yet to be published.	
Glucagon-like peptide (GLP-1)	Neuroprotective [55]	Intravitreal injections of exedin-4 (a GLP-1 analogue) prevent ERG abnormalities in rats with streptozotoin-induced diabetes [56]. Topical administration of GLP-1R agonists prevents retinal neurodegeneration in mice with diabetes [57].	2 large clinical trials of GLP-1 analogues in type 2 diabetics with high cardiovascular risk (LEADER and SUSTAIN-6) have shown neutral benefit [58] or even worsening of DR compared to placebo [59]. However, these studies were not designed to assess progression of DR.
Doxycycline	Anti-inflammatory and neuroprotective [60]	Low-dose oral doxycycline improves inner retinal function in DR compared to placebo [61].	Although statistical significance was achieved at multiple time points, it was a small, proof-of-concept trial.
Interleukin 1β (IL-1β)	Inflammatory cytokine	Systemic IL-1β inhibition has been shown to stabilize retinal neovascular changes in proliferative DR and reduce macular edema [62].	Open-label, small, prospective pilot study. Reduction in macular edema was not statistically significant.
Tumor necrosis factor α (TNF-α)	Inflammatory, induces vascular changes	Intravitreal injection of TNF-α inhibitor decreased capillary degeneration in diabetic rats [63].	Very small study in rats, with other primary endpoints.

In addition to finding new targets to treat earlier stages of DR, research is being conducted using nanoparticles to allow for sustained delivery of drug, as well as alternative (topical) drug delivery systems. Nanotechnology is currently being applied to anti-VEGF medications, and several other new mediators of inflammation and angiogenesis. Nanoparticles are particularly designed to cross the blood-retinal barrier, thereby allowing for better penetration into the retina [50]. These methods remain under development and clinical application appears to remain in the future for DR.

If treating DR in earlier stages is to truly become a common clinical practice, though, new diagnostic techniques are needed to identify changes before they are visible on exam and to track response to treatment. Circulating biomarkers and new imaging modalities are being investigated to use as clinical indicators and new endpoints for clinical trials. Inflammatory cytokines are most often reported as circulating biomarkers associated with early DR. Three inflammatory mediators in particular: interleukin 6 (IL-6), tumor necrosis factor α (TNF-α), and C-reactive protein (CRP), when combined in a z-score, are associated with development of retinopathy, nephropathy and cardiovascular disease in diabetics [51]. Inflammatory and vasoactive mediators produced and measured locally, in intraocular fluids, have also been identified. An increase in glial fibrillary acidic protein (GFAP), for example, is noted in the aqueous humor of patients with diabetes and no signs of DR or with non-proliferative DR compared to age-matched healthy controls [52]. Challenges remain, however, in finding biomarkers specific to DR that are also easily/non-invasively measured. As such, new imaging techniques combined with a better understanding of biomarkers is promising for developing better diagnostic tools for early disease. Frimmel et al. recently reported a technique in which imaging probes were developed to target a specific endothelial surface molecule known to participate in breakdown of the blood-retinal barrier in DR (ICAM-1). This probe allowed for visualization of the expression of ICAM-1 on the endothelial surface in vivo in rats. Increased visibility of the probe was noted on imaging from diabetic animals compared to controls [53], suggesting that synergistic development of biomarkers and imaging technology will allow for detection of early DR in the future.

A projected tripling in the prevalence of DR in the next several decades, however, cannot be effectively managed with new diagnostic and treatment options alone. Changes in health care delivery paradigms will also be required. Similar to the recent shift towards team-based approaches for cancer, diabetes will need to be approached in a more comprehensive manner. Close collaboration between ophthalmologists, endocrinologists, nephrologists, nutritionists, social workers, and all others involved in the care of a diabetic patient will be imperative for an efficient use of community and health system resources, and to optimize outcomes for individual patients.

## Conclusion

The immense improvements in the care of patients with diabetes and DR over the past 50 years are an example of the significant impact laboratory and clinical research can make on the management of chronic systemic illness. Despite great advances, though, the projected increase in the number of patients with DR in the coming decades reminds us that there is still progress to be made. Current research leading to a better understanding of molecular pathways, development of novel therapeutic targets, and use of nanotechnology, coupled with constantly improving diagnostic and imaging technology and collaborative health care delivery systems, promises to further our ability to enhance and maintain vision in diabetic patients.

## Abbreviations

CRP: C-reactive protein
DCCT: Diabetes Control and Complications Trial
DR: Diabetic retinopathy
GFAP: Glial fibrillary acidic protein
GLP-1: Glucagon-like peptide
IL-1β: Interleukin 1β
IL-6: Interleukin 6 (IL-6)
OCT: Optical coherence tomography
PRP: Pan-retinal photocoagulation
TNF-α: Tumor necrosis factor α
UKPDS: United Kingdom Prospective Diabetes Study
VEGF: Vascular endothelial growth factor
WESDR: Wisconsin Epidemiologic Study of Diabetic Retinopathy
